# Prevalence and Impact of Preexisting Comorbidities on Overall Clinical Outcomes of Hospitalized COVID-19 Patients

**DOI:** 10.1155/2022/2349890

**Published:** 2022-04-06

**Authors:** Rajeswari Koyyada, Balakrishna Nagalla, Anusha Tummala, Anula D. Singh, Sreekanth Patnam, Ravikiran Barigala, Mahati Kandala, Vamsi Krishna, Sasidhar V. Manda

**Affiliations:** ^1^Apollo Hospitals Educational and Research Foundation (AHERF), Apollo Hospitals, 500096, India; ^2^Department of Biostatistics, Apollo Institute of Medical Sciences and Research (AIMSR), 500033, India; ^3^Indian Institute of Technology Hyderabad (IITH), Hyderabad 502285, India; ^4^Department of Infectious Diseases, Apollo Health City, 500033, India; ^5^Urvogelbio Private Ltd., Apollo Hospitals Health City, 500033, India

## Abstract

COVID-19 risk increases with comorbidities, and the effect is magnified due to the contribution of individual and combined comorbidities to the overall clinical outcomes. We aimed to explore the influence of demographic factors, clinical manifestations, and underlying comorbidities on mortality, severity, and hospital stay in COVID-19 patients. Therefore, retrospective chart reviews were performed to identify all laboratory-confirmed cases of SARS-CoV-2 infection in Apollo Hospitals, Hyderabad, between March 2020 and August 2020.A total of 369 confirmed SARS-CoV-2 cases were identified: 272 (73.7%) patients were male, and 97 (26.2%) were female. Of the confirmed cases, 218 (59.1%) had comorbidities, and 151 (40.9%) were devoid of comorbidities. This study showed that old age and underlying comorbidities significantly increase mortality, hospital stay, and severity due to COVID-19 infection. The presence of all four comorbidities, diabetes mellitus (DM) + Hypertension (HTN) + coronary artery disease (CAD) + chronic kidney disease (CKD), conferred the most severity (81%). The highest mortality (OR: 44.03, 95% CI: 8.64-224.27) was observed during the hospital stay (12.73 ± 11.38; 95% CI: 5.08-20.38) in the above group. Multivariate analysis revealed that nonsurvivors are highest (81%) in (DM + HTN + CAD + CKD) category with an odds ratio (95% CI) of 44.03 (8.64-224.27). Age, gender, and comorbidities adjusted odds ratio decreased to 20.25 (3.77-108.77). Median survival of 7 days was observed in the (DM + HTN + CAD + CKD) category. In summary, the presence of underlying comorbidities has contributed to a higher mortality rate, greater risk of severe disease, and extended hospitalization periods, hence, resulting in overall poorer clinical outcomes in hospitalized COVID-19 patients.

## 1. Introduction

Novel coronavirus disease (COVID-19), a global disorder caused by severe acute respiratory syndrome coronavirus 2 (SARS-CoV-2), was initially reported in December 2019 in Wuhan city of China. It had rapidly spread across over 180 countries and was declared a pandemic by WHO in March 2020 [1]. This pandemic had an enormous adverse impact on socioeconomic conditions, traditional human lifestyle, and healthcare resources worldwide. As of August 2021, 213,752,662 confirmed cases of COVID-19 and 4,459,381 deaths were recorded globally [2]. As the virus continues to evolve, more infections and mortality are expected worldwide. On the other hand, recurrence of COVID-19 infection has also been reported despite the ongoing global vaccination drives [3]. Therefore, it is essential to understand the clinical and epidemiological characteristics of COVID-19 infected patients to develop effective preventative strategies to stall the spread of infection.

Although evidence from recent studies suggests that individuals with preexisting comorbidities are at a greater risk of mortality due to COVID-19 [4], the available data regarding the association between COVID-19 and underlying comorbidities is still limited. The most prevalent comorbidities in COVID-19 patients include diabetes, hypertension, cardiovascular disease, renal complications, and cancer. Although all comorbidities do not confer the same risk, many of these are strongly associated with each other, resulting in multiple comorbid conditions in many patients, putting them at a greater risk of severity and mortality associated with COVID-19 [5]. It was also reported that poorer prognosis and clinical outcomes were observed in patients with any comorbidity or a combination of comorbidities than those without [6].

In COVID-19 patients, hypertension was reported to be the highest preexisting comorbidity with an increased risk for severe infection and death [7–10]. An increased mortality rate has also been highly reported in patients with underlying cardiovascular disease [6, 9, 11]. At the same time, diabetes stands as the third most prevalent comorbidity [12, 13] and is responsible for developing severe illness in COVID-19 patients [14]. Chronic kidney disease is associated with disease severity and increased mortality in COVID-19 patients [15]. However, the prevalence of comorbidities was highly variable in COIVD-19 patients, as reported in many studies [14].

In our study, we have presented the baseline demographics, clinical parameters, prevalence, and impact of the four most prevalent comorbidities, diabetes, hypertension, cardiovascular disease, and chronic kidney disease, on the severity of the disease and overall outcome in hospitalized COVID-19 patients treated at our hospital based in the state of Telangana. In addition, we have reported the impact of these comorbidities individually and as combinations that will be at play.

## 2. Materials and Methods

### 2.1. Data Collection

Apollo Hospitals was established in 1983 and is India's first corporate hospital network with 74 hospitals, pan-India with 12,000 beds. The study cohort consists of 369 patients admitted and treated at Hyderabad, Telangana, India. This is a 550 bedded multispeciality tertiary clinic with 50 specialities and superspecialities. The study is from patients admitted in the 1^st^ wave from March to August 2020, with SARS-CoV-2 infection confirmed by reverse transcriptase-polymerase chain reaction (RT-PCR) test on nasopharyngeal and oropharyngeal swabs. This retrospective study was approved by The Institutional Ethics Committee (Biomedical Research (IEC-BMR), Apollo Hospitals) with IEC Application no: AHJ-ACD-071/08-2, and the study protocol no: CMBRC/2021/007. Details of physical examination and vital parameters such as age, blood pressure, pulse, temperature, rate of respiration, oxygen saturation (SPO2), clinical symptoms including fever, cough, cold, sore throat, shortness of breath, weakness, diarrhea, etc. were recorded at the time of admission. In addition, all comorbidities were recorded in the patient case sheet. In our study, we have highlighted the role of four selected comorbidities, diabetes mellitus (DM), hypertension (HTN), coronary artery disease (CAD), and chronic kidney disease (CKD), both individually as well as combined risk factors for the severity and overall outcome of the disease.

### 2.2. Statistical Analysis

Patient data were recorded and analyzed using statistical software, GraphPad PRISM (version 9) and SPSS (version 24). Categorical variables were analyzed using the Chi-square test (to study the association). Frequency, mean, and standard deviation were determined using a descriptive test. Continuous variables were compared using *t*-test and one-way ANOVA with post hoc test of LSD. Survival curves were drawn using Kaplan-Meier survival plot analysis and compared using log-rank tests. Multivariate analysis of overall survival with age, gender, and comorbidities was performed using logistic regression analysis. *p* values less than 0.05 were considered statistically significant.

## 3. Results and Discussion

### 3.1. Baseline Clinical Characteristics

A total of 369 patients were recruited for this study, including 151 (40.9%) patients with no comorbidities and 218 (59.1%) patients with one or more comorbidities. The sex ratio was 272 (73.7%) male and 103 (26.2%) female. The overall mean age of the study population was 54.48 ± 17.09 years, while the mean age of patients without any comorbidity was 43.61 ± 16.55 years, and with any comorbidity was 62.13 ± 12.81 years. The mean age of patients in any comorbidity group was significantly higher when compared to the no comorbidity group (*p* < 0.05). Clinical parameters such as systolic blood pressure (*p* ≤ 0.001), SPO2 (*p* = 0.005), and respiration rate (*p* = 0.001) were also significantly different between no-comorbidity and any comorbidity groups. 117 (31.7%) of the study population were admitted to intensive care units (ICU) with a requirement of supplemental oxygen supply. The remaining 252 (68.3%) of the population were admitted to non-ICU wards. Among the ICU admissions, 23 (19.7%) patients had no underlying comorbidities, and 94 (80.3%) had at least one comorbidity. Significantly greater number of ICU admissions was observed in patients with comorbidities than without (*p* ≤ 0.001). The presence of comorbidity significantly correlated (*p* ≤ 0.001) with the length of hospital stay (number of days of hospitalization). Demographic and baseline clinical characteristics of all patients and comparison between no-comorbidity vs. any-comorbidity groups are presented in Table 1.

### 3.2. Prevalence of Comorbidities in the Study Cohort

218 (59.1%) patients in the study cohort had at least one comorbidity. Although all comorbidities were recorded, we have selected only four (most prevalent) comorbidities for our analysis, namely, diabetes mellitus (DM) (11.9%), hypertension (HTN) (8.1%), coronary artery disease (CAD) (1.9%), and chronic kidney disease (CKD) (0.8%). We then stratified patients based on single and multiple comorbidities. The combinations of comorbidities derived were DM + HTN (18.2%), DM + CAD (1.1%), DM + CKD (0.3%), HTN + CAD (2.4%), HTN + CKD (0.3%), DM + HTN + CAD (7.9%), DM + HTN + CKD (3.0%), HTN + CAD + CKD (0.3%), and DM + HTN + CAD + CKD (3.0%). However, three groups (DM + CKD, HTN + CKD, and HTN + CAD + CKD) were excluded from further analysis due to an insufficient sample number. The combination of DM and HTN was the most prevalent (18.2%) of all comorbidities analyzed. We have also analyzed the impact of multiple comorbidities alongside the above categories. 22.8% of the cohort had one comorbidity, 22.2% consisted of any two comorbidities, 11.1% and 3.0% of patients had any three and all four comorbidities, respectively. The distribution of comorbidities is presented in Tables 2 and 3.

### 3.3. Impact of Comorbidities on Overall Clinical Outcomes

**Figure 1:**
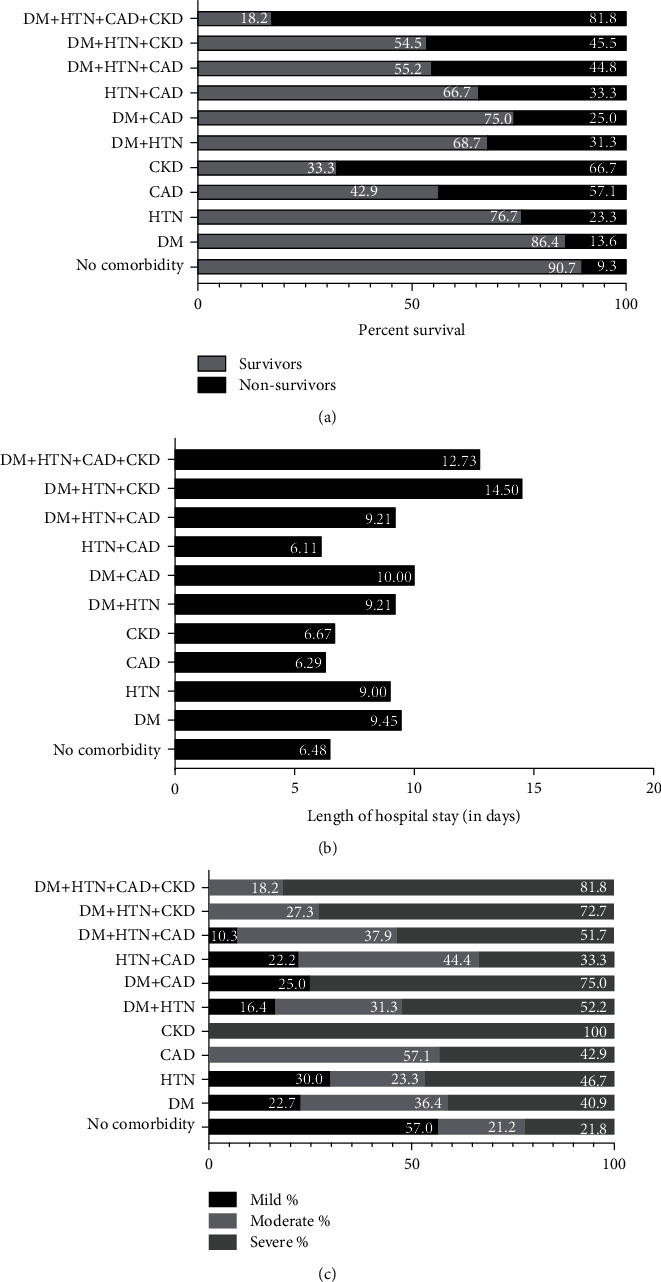
Impact of comorbidities on overall clinical outcomes. (a) represents the percent survival, (b) shows the length of hospital stay, and (c) represents disease severity in hospitalized COVID-19 patients with or without preexisting comorbidities.

Survival was highest (90.7%) in patients with no comorbidities, and the highest percentage (81.8%) of nonsurvivors was observed in the DM + HTN + CAD + CKD group (OR = 44.03, 95%CI = 8.64 − 224.27). A significant association between mortality and presence of comorbidities was observed in all groups including HTN (OR (95% CI)) (2.97 (1.08-8.17)), CAD (7.33 (1.49-36.16)), CKD (19.57 (1.66-229.6)), DM + HTN (4.46 (2.10-9.49)), DM + HTN + CAD (7.95 (3.18-19.86)), DM + HTN + CKD (8.15 (2.20-30.16)), DM + HTN + CAD + CKD (44.03 (8.64-224.27)), HTN + CAD (4.89 (1.10-21.73)), except DM and DM + CAD groups (*p* > 0.05) as represented in Table 4 (Figure 1(a)). The average span of the hospital stay was significantly longer in DM (95% CI; *p* value) (7.81-11.10; 0.006), HTN (6.58-11.42; 0.044), DM + HTN (7.77-10.65; 0.003), DM + HTN + CAD (6.21-11.79; 0.032), DM + HTN + CKD (7.61-21.39; ≤0.001), and DM + HTN + CAD + CKD (5.08-20.38; 0.001) groups in comparison to the no-comorbidity group as shown in Table 4 and depicted in Figure 1(b). The severity of the disease is higher in CKD (100%) and DM + HTN + CAD + CKD (81.8%) groups. Disease severity was significantly high in the presence of all four comorbidities, with p≤0.001, as shown in Table 5 and Figure 1(c), except in HTN, CAD, and HTN + CAD groups (severity of disease was not compared between the comorbidity groups).

### 3.4. Number of Comorbidities and Their Impact on Overall Clinical Outcomes

**Figure 2:**
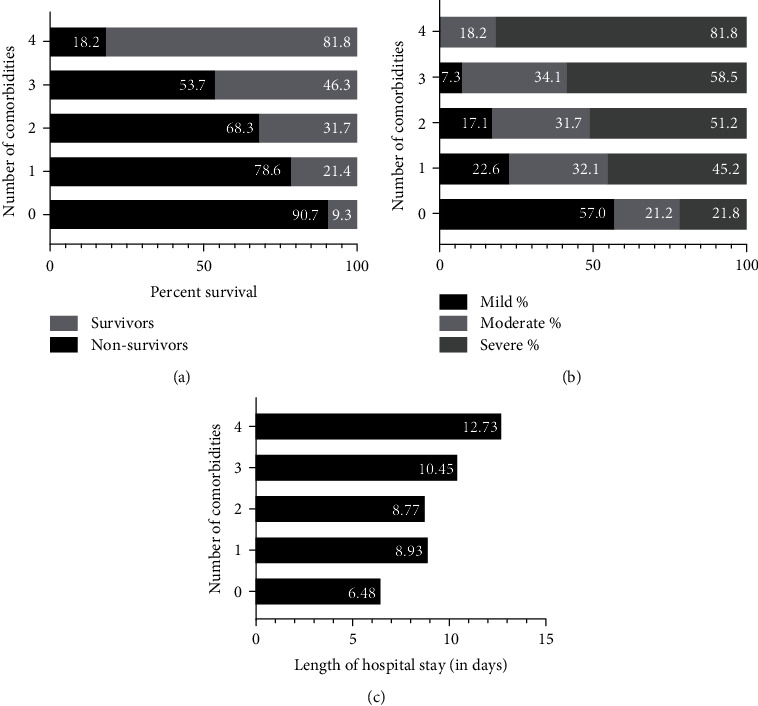
Impact of number of comorbidities on overall clinical outcomes. (a) represents the percent survival, (b) shows disease severity, and (c) represents the length of hospital stay in hospitalized COVID-19 patients with or without multiple comorbidities.

With an increase in the number of comorbidities, mortality also increased significantly. The percentage of nonsurvivors was least 9.3% (*p* ≤ 0.001) in the no-comorbidity group, 21.4% (*p* = 0.011 in patients having at least one comorbidity, 31.7% (*p* ≤ 0.001) with any two comorbidities, 46.3% (*p* ≤ 0.001) with any three comorbidities, and 81.8% (*p* ≤ 0.001) with all four comorbidities. The mean length of hospital stay in patients with no underlying comorbidity was 6.5 days (95%CI = 5.55 − 7.40), 8.9 days (95%CI = 7.68 − 10.18) in patients with one comorbidity, 8.7 days (95%CI = 7.50 − 10.04) with any two comorbidities, 10.4 days (95%CI = 7.87 − 13.03) with any three comorbidities, and 12.7 days (95%CI = 5.08 − 20.38) with all four comorbidities. A significant (*p* ≤ 0.001) increasing trend in the number of days of hospital stay was observed in patients with an increasing number of comorbidities. The severity of the disease was highest (81.8%) in patients with four comorbidities and lowest (21.8%) in patients with no underlying comorbidities with a significance of *p* ≤ 0.001. Greater risk of mortality, extended hospitalization period, and severe disease was observed in COVID-19 patients with an increase in the number of underlying comorbidities. The above observations are presented in Table 6 and Figure 2.

### 3.5. Correlation of Age, Gender, and Comorbidities with Mortality

Logistic regression models were applied to comorbidities, age, gender, and overall survival of COVID-19 patients. Multivariate analysis revealed a decrease in odds ratio, suggesting a reduced mortality risk upon correlating with age, gender, and comorbidity in all groups, as shown in Table 7. After adjusting for age and gender, the decreased mortality risk could be due to our uneven distribution pattern of the selected study population. A significant association (*p* < 0.05) with mortality was observed for HTN (OR (95% CI)) (2.97 (1.08-8.17)), CAD (7.33 (1.49-36.16)), CKD (19.57 (1.66-229.6)), DM + HTN (4.46 (2.10-9.49)), DM + HTN + CAD (7.95 (3.18-19.86)), DM + HTN + CKD (8.15 (2.20-30.16)), and DM + HTN + CAD + CKD (44.03 (8.64-224.27)) groups. However, after adjusting for age and gender, only four combinations of comorbidities, DM + HTN (AOR (95% CI)) (2.70 (1.20-6.07)), DM + HTN + CAD (4.16 (1.55-11.17)), DM + HTN + CKD (5.09 (1.26-20.45)), and DM + HTN + CAD + CKD (20.25 (3.77-108.77)) showed significant association with mortality (*p* < 0.05).

### 3.6. Survival Curves

**Figure 3:**
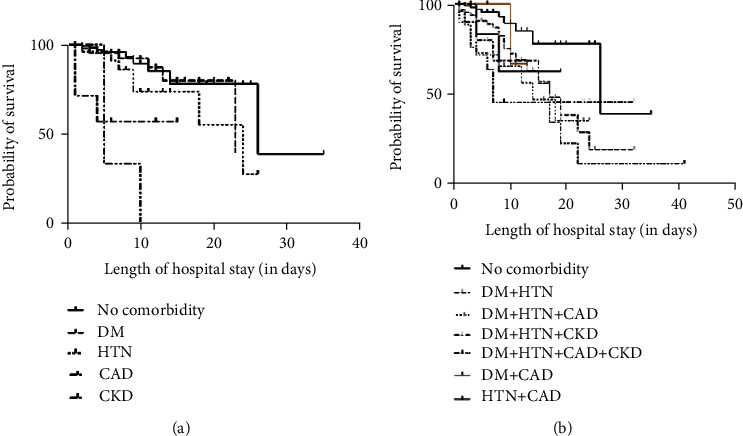
Survival curves. (a) Comparison of probability of survival between no-comorbidity and individual comorbidities. (b) Combination of comorbidities.

Survival curves were plotted using Kaplan-Meier survival plot analysis for all comorbidity groups and no comorbidity group. The median survival of patients with no comorbidity was 26 days, and the median survival of 5 days was observed in the CKD group, as represented in Table 8. Log-rank median survival test is significant (*p* < 0.0001). Median survival among the combination groups includes (DM + HTN + CKD + CAD) < (HTN + CAD) < (DM + CAD) < (DM + HTN + CAD) < (DM + HTN + CKD) < (DM + HTN) < (no comorbidity), *p* = 0.0054, as shown in Figure 3. Furthermore, a significant negative association of survival with comorbidities was observed with *p* < 0.05, as shown in Table 9.

## 4. Discussion

In this study, we report the impact of four selected comorbidities, DM, HTN, CAD, and CKD, at the individual level and their combinations, on mortality, severity of the disease, and length of hospital stay of COVID-19 patients.

In the baseline clinical characteristics, we observed significantly high systolic blood pressure, respiratory rate, mean age of patients, and low SPO2 in patients having one or more comorbidities than those without. In addition, a considerably longer length of hospital stay was observed in patients with any comorbidity, suggesting that patients with underlying comorbidities were required to spend more treatment days in the hospital when compared to patients without any comorbidity. There were fewer survivors among those with comorbidities. As reported elsewhere, patients with two or more comorbidities had poorer outcomes [16]. Mortality was significantly high in patients with HTN, CAD, CKD, and all combinations analyzed except DM and DM + CAD groups compared to patients without comorbidities. In our study, DM was not independently associated with mortality. However, the risk of progression to severe disease was observed in patients with DM, as has been highlighted in previous studies [17, 18].

The presence of comorbidities is significantly associated with the severity of the disease in patients having DM, CKD, DM + HTN, DM + CAD, DM + HTN + CAD, DM + HTN + CKD, DM + HTN + CAD + CKD, which is evident through Table 5. Furthermore, our results show an association between the length of hospital stay and the presence of underlying comorbidities as the duration of hospital stay was significantly longer in patients with DM, HTN, DM + HTN, DM + HTN + CAD, DM + HTN + CKD, and DM + HTN + CAD + CKD compared to patients without any comorbidities. The Kaplan-Meier plots showed that for individual comorbidities, the relative probability of mortality was CAD > CKD > DM > HTN > no comorbidity, *p* < 0.0001. With comorbidities, the least survival was observed in the (DM + HTN + CKD + CAD) group. The mean time from the onset of symptoms to death differs from the published information inferring differences in arrival time, stage of patients arrival to the hospital, ABO blood groups, probably ethnicity, etc.

Our analysis indicates that an increasing number of comorbidities has significantly affected clinical outcomes in hospitalized COVID-19 patients. The mortality rate increased with the number of comorbidities in our study, consistent with previously reported studies [16, 19]. Likewise, a prolonged hospitalization period and a higher risk of severe disease were observed in patients with an increasing number of comorbidities compared to those with none. However, no significance was observed in the length of the hospital stay in patients having one or two or three or four comorbidities. In addition, disease severity was not significantly different in patients with one or two and three comorbidities as indicated by variation in superscripts, as shown in Table 6.

After adjusting for age, gender, and comorbidities in multivariate analysis, the risk of mortality was reduced with HTN, CAD, and CKD. Combinations of comorbidities, DM + HTN (OR: 2.70, *p* = 0.016), DM + HTN + CAD (OR: 4.16, *p* = 0.005), DM + HTN + CKD (OR: 5.09, *p* = 0.022), and DM + HTN + CAD + CKD (OR: 20.25, *p* ≤ 0.001) were identified as independent risk factors of mortality in hospitalized COVID-19 patients. In summary, our findings agree with the published studies on the effects of comorbidities [20].

Studies have reported comparative differences in clinical outcomes in COVID-19 patients during 1^st^ and 2^nd^ waves of infection in India and worldwide [21]. Several variations in clinical characteristics in both waves in India were reported, including a low death rate and patients less affected by comorbidities in the second wave than the first [22]. Another study reported a sharp decline in the case of fatality rate in the second wave from the first wave in India, which could be due to the younger demographic profile [23].

We observed variations in outcomes over time in our study setting by comparing present data with data collected from hospitalized COVID-19 patients admitted to our hospital during the postsecond wave of infection in India, i.e., from August 2021 to December 2021. In this new cohort, comorbidities did not show a significant association with mortality and severity of the disease, unlike that of present data. However, patients with comorbidities required longer hospitalization than those without in both the data sets. This variation in outcomes over time could be attributed to the emergence of new variants/mutants of the virus, improvement in overall understanding of the disease management such as evolving therapeutic regimes, preparedness in healthcare settings, and most importantly, the implementation of mass vaccination drives. Our observations are consistent with other studies that reported both similarities [24] and variations [21] in their data over a period of time. All the above observations are presented in Supplementary Tables 1-4.

Although several studies have reported the impact of comorbidities on the clinical outcomes in COVID-19 patients, our study highlights the prevalence and impact of all possible combinations of the four most prevalent comorbidities, DM, HTN, CAD, and CKD, on the overall outcomes in hospitalized COVID-19 patients which were not reported earlier.

Limitations of our work, the study population included patients from a single centre within Hyderabad city, India. The presence of comorbidities was confirmed with patient medical records only. However, the degree of control and timespan of comorbidities and interaction were not considered, which might affect the outcomes. Relatively low sample numbers in groups such as CAD, CKD, DM + CAD, and HTN + CAD could also affect the results. Furthermore, the study is limited to the initial four months of India's first wave of infection, from March 2020 to August 2020.

## 5. Conclusions

We consider this study to be an essential contribution to the worldwide effort to understand the role of comorbidities (single and multiple) in the outcome of COVID-19 patients; we have presented significant aspects of COVID-19 prognosis, including the epidemiological profile, length of hospital stay, survival, mortality, and baseline comorbidities. The critical findings of our study are that a combination of DM + HTN, DM + HTN + CAD, DM + HTN + CAD, and DM + HTN + CAD + CKD are essential risk factors to be considered while managing COVID-19 patients' hospital stay, independent of age and gender. Our results imply that both the category and the number of comorbidities should be regarded as the prognosis in COVID-19 patients. With evolving strains, knowledge of the effects of these comorbidities, independent of age and gender, is instrumental primarily to protect individuals with conditions that increase adverse outcomes from COVID-19.

## Figures and Tables

**Table 1:** Demographics and baseline clinical characteristics of patients hospitalized with COVID-19. Data expressed as mean ± SD. *p* value less than 0.05 was considered statistically significant.

Variable	Total	No comorbidity (*N*, % within total)	Any comorbidity (*N*, % within total)	*p* value
Study size	369	151 (40.9%)	218 (59.1%)	
Male∗	272 (73.7%)	103 (37.8%)	169 (62.1%)	0.051
Female∗	97 (26.2%)	48 (49.4%)	49 (50.5%)	
Age (in years)	54.48 ± 17.09	43.61 ± 16.55	62.13 ± 12.81	≤0.001
Blood pressure-systolic (mmHg)	123.53 ± 18.86	118.20 ± 12.58	127.09 ± 21.38	≤0.001
Blood pressure-diastolic (mmHg)	76.73 ± 10.76	75.67 ± 10.12	77.44 ± 11.13	0.133
Pulse rate (beats/minute)	91.53 ± 18.92	89.92 ± 18.06	92.64 ± 19.44	0.184
Respiratory rate (breaths/minute)	24.31 ± 7.09	22.89 ± 6.98	25.31 ± 7.02	0.001
SPO2 (%)	93.55 ± 10.08	95.67 ± 5.81	92.07 ± 12.00	0.005
Temperature (∗Fahrenheit)	98.69 ± 1.57	98.56 ± 1.37	98.78 ± 1.70	0.201
Survivors∗	283 (76.69%)	137 (48.4%)	146 (51.5%)	≤0.001
Nonsurvivors∗	86 (23.30%)	14 (16.3%)	72 (83.7%)	
ICU admissions∗	117 (31.7%)	23 (19.7%)	94 (80.3%)	≤0.001
Non-ICU admissions∗	252 (68.3%)	125 (49.6%)	127 (50.4%)	
Length of hospital stay (in days)	8.16 ± 6.50	6.13 ± 5.77	9.4 ± 6.64	≤0.001

Note: ∗categorical variables were analyzed using Chi-square test.

**Table 2:** Prevalence of comorbidities and multiple morbidities in study population.

No.		Frequency	%
1	No comorbidity	151	40.9
2	DM	44	11.9
3	HTN	30	8.1
4	CAD	7	1.9
5	CKD	3	0.8
6	DM + HTN	67	18.2
7	DM + CAD	4	1.1
8	DM + CKD	1	0.3
9	HTN + CAD	9	2.4
10	HTN + CKD	1	0.3
11	DM + HTN + CAD	29	7.9
12	DM + HTN + CKD	11	3.0
13	HTN + CAD + CKD	1	0.3
14	DM + HTN + CAD + CKD	11	3.0
	Total	369	100.0

**Table 3:** Distribution of multiple morbidities in study population.

No.		Frequency	%
0	No morbidity	151	40.9
1	One morbidity	84	22.8
2	Two morbidity	82	22.2
3	Three morbidity	41	11.1
4	Four morbidity	11	3.0
	Total	369	100.0

**Table 4:** Overall survival and length of hospital stay in hospitalized COVID-19 patients with or without single and multiple comorbidities. Length of hospital stay data is represented as mean ± SD.

Variable	Total (*N*)	Survivors (*N*, % within total)	Nonsurvivors (*N*, % within total)	Odds ratio (95% CI)	*p* value	Length of hospital stay (in days)	95% CI	*p* value
No comorbidity∗	151	137 (90.7)	14 (9.3)		≤0.001	6.48 ± 5.75	5.55-7.40	
DM	44	38 (86.4)	6 (13.6)	1.54 (0.55-4.29)	0.404	9.45 ± 5.40	7.81-11.10	0.006
HTN	30	23 (76.7)	7 (23.3)	2.97 (1.08-8.17)	0.034	9.00 ± 6.49	6.58-11.42	0.044
CAD	7	4 (57.1)	3 (42.9)	7.33 (1.49-36.16)	0.014	6.29 ± 5.34	1.34-11.23	0.937
CKD	3	1 (33.3)	2 (66.7)	19.57 (1.66-229.6)	0.018	6.67 ± 2.88	-0.50-13.84	0.959
DM + HTN	67	46 (68.7)	21 (31.3)	4.46 (2.10-9.49)	≤0.001	9.21 ± 5.88	7.77-10.65	0.003
DM + HTN + CAD	29	16 (55.2)	13 (44.8)	7.95 (3.18-19.86)	≤0.001	9.21 ± 6.88	6.21-11.79	0.032
DM + HTN + CKD	11	6 (54.5)	5 (45.5)	8.15 (2.20-30.16)	0.002	14.50 ± 10.24	7.61-21.39	≤0.001
DM + HTN + CAD + CKD	11	2 (18.2)	9 (81.8)	44.03 (8.64-224.27)	≤0.001	12.73 ± 11.38	5.08-20.38	0.001
DM + CAD	4	3 (75.0)	1 (25.0)	3.26 (0.31-33.49)	0.320	10.00 ± 2.94	5.32-14.68	0.267
HTN + CAD	9	6 (66.7)	3 (33.3)	4.89 (1.10-21.73)	0.037	6.11 ± 5.68	1.74-10.48	0.865

Note: ∗no comorbidity is the reference group.

**Table 5:** Severity of disease in hospitalized COVID-19 patients.

Variable	Total (*N*, %)	Mild (*N*, % within total)	Moderate (*N*, % within total)	Severe (*N*, % within total)
No morbidity	151	86 (57.0)	32 (21.2)	33^a^ (21.9)
DM	44	10 (22.7)	16 (36.4)	18^b^ (40.9)
HTN	30	9 (30.0)	7 (23.3)	14^a^ (46.7)
CAD	7	0 (0.0)	4 (57.1)	3^a^ (42.9)
CKD	3	0 (0.0)	0 (0.0)	3^b^ (100.0)
DM + HTN	67	11 (16.4)	21 (31.3)	35^b^ (52.2)
DM + HTN + CAD	29	3 (10.3)	11 (37.9)	15^b^ (51.7)
DM + HTN + CKD	11	0 (0.0)	3 (27.3)	8^b^ (72.7)
DM + HTN + CAD + CKD	11	0 (0.0)	2 (18.2)	9^b^ (81.8)
DM + CAD	4	1 (25.0)	0 (0.0)	3^b^ (75.0)
HTN + CAD	9	2 (22.2)	4 (44.4)	3^a^ (33.3)
*p* value				≤0.001

Notes: *p* ≤ 0.001 suggests significant association between comorbidity groups and severity of the disease. *t*-test was viewed for comparison of proportions between no comorbidity and other comorbidity groups. Variations in superscripts (a, b) indicate significance (*p* < 0.05) of disease severity across these groups.

**Table 6:** Overall survival, length of hospital stay, and severity of the disease in COVID-19 patients with multiple comorbidities.

Number of comorbidities	Total (N)	Survivors (*N*, % within total)		Nonsurvivors (*N*, % within total)	*p* value	Length of hospital stay (in days)	95% CI	Mild (*N*, % within total)	Moderate (*N*, % within total)	Severe (*N*, % within total)
0	151	137 (90.7)		14 (9.3)	≤0.001	6.48^a^ ± 5.75	5.55-7.40	86 (57.0)	32 (21.2)	33^a^ (21.8)
1	84	66 (78.6)		18 (21.4)	0.011	8.93^b^ ± 5.74	7.68-10.18	19 (22.6)	27 (32.1)	38^b^ (45.2)
2	82	56 (68.3)		26 (31.7)	≤0.001	8.77^b^ ± 5.79	7.50-10.04	14 (17.1)	26 (31.7)	42^bc^ (51.2)
3	41	22 (53.7)		19 (46.3)	≤0.001	10.45^b^ ± 8.17	7.87-13.03	3 (7.3)	14 (34.1)	24^bc^ (58.5)
4	11	2 (18.2)		9 (81.8)	≤0.001	12.73^b^ ± 11.38	5.08-20.38	0 (0.0)	2 (18.2)	9^c^ (81.8)
*p* value						≤0.001				≤0.001

**Table 7:** Multivariate analysis of overall survival correlated with age, gender, and comorbidities in COVID-19 patients.

Variable	Total, *N*	Survivors, *N* (% within total)	Nonsurvivors *N* (% within total)	Adjusted ∗			
				Odds ratio (95% CI)	*p* value	Odds ratio (95% CI)	*p* value
No morbidity	151	137 (90.7)	14 (9.3)		≤0.001		0.013
DM	44	38 (86.4)	6 (13.6)	1.54 (0.55-4.29)	0.404	1.20 (0.41-3.49)	0.737
HTN	30	23 (76.7)	7 (23.3)	2.97 (1.08-8.17)	0.034	2.10 (0.73-6.00)	0.166
CAD	7	4 (57.1)	3 (42.9)	7.33 (1.49-36.16)	0.014	3.73 (0.70-19.74)	0.121
CKD	3	1 (33.3)	2 (66.7)	19.57 (1.66-229.6)	0.018	10.64 (0.81-139.9)	0.072
DM + HTN	67	46 (68.7)	21 (31.3)	4.46 (2.10-9.49)	≤0.001	2.70 (1.20-6.07)	0.016
DM + HTN + CAD	29	16 (55.2)	13 (44.8)	7.95 (3.18-19.86)	≤0.001	4.16 (1.55-11.17)	0.005
DM + HTN + CKD	11	6 (54.5)	5 (45.5)	8.15 (2.20-30.16)	0.002	5.09 (1.26-20.45)	0.022
DM + HTN + CAD + CKD	11	2 (18.2)	9 (81.8)	44.03 (8.64-224.27)	≤0.001	20.25 (3.77-108.77)	≤0.001
DM + CAD	4	3 (75.0)	1 (25.0)	3.26 (0.31-33.49)	0.320	2.86 (0.22-36.45)	0.417
HTN + CAD	9	6 (66.7)	3 (33.3)	4.89 (1.10-21.73)	0.037	2.99 (0.60-14.78)	0.179

**Table 8:** Median survival in all comorbidity groups analyzed.

	No comorbidity	DM	HTN	CAD	CKD	DM + HTN	DM + CAD	HTN + CAD	DM + HTN + CAD	DM + HTN + CKD	DM + HTN + CKD + CAD
Median survival	26	23	24	9.5	5	17	11.5	10	14	17	7

**Table 9:** Log-rank test analysis of survival curves.

Comparison of survival curves-log-rank (mantel-Cox) test		
	No comorbidity vs. DM, HTN, CAD, CKD	No comorbidity vs. DM + HTN, DM + CAD, HTN + CAD, DM + HTN + CAD, DM + HTN + CKD, DM + HTN + CAD + CKD
*p* value	0.0054	<0.0001
Chi-square	18.38	33.42
df	6	4

## Data Availability

Request should be placed to the corresponding author.

## Supplementary Materials

Supplementary Materials Supplementary Table 1a: prevalence of comorbidities and multiple morbidities in study population. Supplementary Table 1b: distribution of multiple morbidities in study population. Supplementary Table 2: overall survival and length of hospital stay in hospitalized COVID-19 patients with or without single and multiple comorbidities. Data is represented as mean ± SD. Supplementary Table 3: the severity of disease in hospitalized COVID-19 patients. Supplementary Table 4: overall survival, length of hospital stay, and severity of the disease in COVID-19 patients with multiple comorbidities.
