# Supplementary material for: Prevalence and Impact of Preexisting Comorbidities on Overall Clinical Outcomes of Hospitalized COVID-19 Patients
Source: Biomed Res Int. 2022 Apr 6;2022:2349890. doi: 10.1155/2022/2349890 (PMC8984738; doi:10.1155/2022/2349890)
Supplement: Supplementary Materials — Supplementary Table 1a: prevalence of comorbidities and multiple morbidities in study population. Supplementary Table 1b: distribution of multiple morbidities in study population. Supplementary Table 2: overall survival and length of hospital stay in hospitalized COVID-19 patients with or without single and multiple comorbidities. Data is represented as mean ± SD. Supplementary Table 3: the severity of disease in hospitalized COVID-19 patients. Supplementary Table 4: overall survival, length of hospital stay, and severity of the disease in COVID-19 patients with multiple comorbidities. [file 2349890.f1.docx]

**Supplementary data**

**Data collection:**

We observed variations in clinical outcomes due to COVID-19 infection over time in our hospital setting by comparing data from the 1^st^ wave, March to August 2020 (labelled as Cohort 1), with post-second wave data, i.e., August 2021 to December 2021 (Cohort 2). After EC approval, we collected data from 265 patients admitted to our hospital with confirmed COVID-19 infection. Statistical analyses were performed as described in the methodology section of the paper. We analyzed the impact of individual and combination of selected comorbidities, DM, HTN, CAD and CKD, on overall survival, length of hospital stay and severity of the disease in cohort 2.

**Results & Discussion:**

The frequency distribution of comorbidities in cohort 2 is represented in Supplementary tables 1a and 1b. Groups with sample number ≤1 was excluded from the analysis. No significant association was observed between comorbidities and mortality in cohort 2, unlike cohort 1. We observed an increased hospital stay in patients with multiple comorbidities, DM+HTN+CAD (p=0.014) and DM+HTN+CKD (p=0.001) compared to patients without comorbidities Supplementary table 2. This observation aligns with our cohort 1 data suggesting that patients with underlying comorbidities required prolonged hospitalization periods. Also, less severe disease was observed in patients with comorbidities than those without in cohort 2 compared to cohort one, as shown in table 3. Moreover, multivariate analysis showed that mortality was not associated with age and gender, unlike cohort 1 (data not shown).

Supplementary table 1b. Distribution of multiple morbidities in study population

cv

Supplementary table 1a. Prevalence of comorbidities and multiple morbidities in study population

cv

|  |  | **Frequency** | **%** |
| --- | --- | --- | --- |
| 1 | No Comorbidity | 133 | 50.2 |
| 2 | DM | 18 | 6.8 |
| 3 | HTN | 42 | 15.8 |
| 4 | CAD | 0 | 0 |
| 5 | CKD | 3 | 1.1 |
| 6 | DM+HTN | 48 | 18.1 |
| 7 | DM+CAD | 1 | 0.4 |
| 8 | DM+CKD | 1 | 0.4 |
| 9 | HTN+CAD | 2 | 0.8 |
| 10 | HTN+CKD | 3 | 1.1 |
| 11 | DM+HTN+CAD | 6 | 2.3 |
| 12 | DM+HTN+CKD | 7 | 2.6 |
| 13 | HTN+CAD+CKD | 0 | 0 |
| 14 | DM+HTN+CAD+CKD | 1 | 0.4 |
|  | Total | 265 | 100.0 |

|  |  | **Frequency** | **%** |
| --- | --- | --- | --- |
| 0 | No Morbidity | 133 | 50.2 |
| 1 | ONE Morbidity | 63 | 23.8 |
| 2 | TWO Morbidity | 55 | 20.7 |
| 3 | THREE Morbidity | 13 | 4.9 |
| 4 | FOUR Morbidity | 1 | 0.4 |
|  | Total | 265 | 100.0 |

Supplementary table 2. Overall survival and length of hospital stay in hospitalized COVID-19 patients with or without single and multiple comorbidities. Data is represented as Mean ±SD.

|  | **Variable** | **Total (N)** | **Survivors**  **(N, % within total)** | **Non-Survivors (N, % within total)** | **Odds ratio (95% CI)** | **p value** | **Length of hospital stay**  **(in days)** | **p value** |
| --- | --- | --- | --- | --- | --- | --- | --- | --- |
|  | No comorbidity* | 133 | 129 (97.0) | 4 (3.0) |  |  | 3.11±2.47 |  |
|  | DM | 18 | 18 (100.0) | 0 (0.0) | 50092243.86 (0.000) | 0.999 | 3.28±1.99 | 0.792 |
|  | HTN | 42 | 40 (95.2) | 2 (4.8) | 0.620 (0.11-3.51) | 0.589 | 3.40±2.83 | 0.507 |
|  | CKD | 3 | 3 (100.0) | 0 (0.0) | 50092243.85 (0.000) | 0.999 | 5.33±0.57 | 0.127 |
|  | DM+HTN | 48 | 44 (91.7) | 4 (8.3) | 0.341 (0.08-1.42) | 0.140 | 2.58±1.98 | 0.207 |
|  | DM+HTN+CAD | 6 | 5 (83.3) | 1 (16.7) | 0.155 (0.01-1.65) | 0.123 | 5.67±5.00 | **0.014** |
|  | DM+HTN+CKD | 7 | 6 (85.7) | 1 (14.3) | 0.186 (0.01-1.93) | 0.159 | 6.43±1.90 | **0.001** |
|  | HTN+CAD | 2 | 2 (100.0) | 0 (0.0) | 50092243.85 (0.000) | 1.000 | 1.00±0.00 | 0.234 |
|  | HTN+CKD | 3 | 3 (100.0) | 0 (0.0) | 50092243.85 (0.000) | 0.999 | 4.33±3.05 | 0.401 |
|  | Total | 262 |  |  |  |  |  |  |

Supplementary Table 3. The severity of disease in hospitalized COVID-19 patients

|  | **Variable** | **Total (N)** | | **Mild (N, % within total)** | | **Moderate (N, % within total)** | **Severe (N, % within total)** |
| --- | --- | --- | --- | --- | --- | --- | --- |
|  | No Morbidity | 133 | | 72 (54.1) | | 40 (30.1) | 21 (15.8) |
|  | DM | 18 | | 5 (27.8) | | 12 (66.7) | 1 (5.6) |
|  | HTN | 42 | | 23 (54.8) | | 11 (26.2) | 8 (19.0) |
|  | CKD | 3 | | 0 (0.0) | | 2 (66.7) | 1 (33.3) |
|  | DM+HTN | 48 | | 21 (43.8) | | 17 (35.4) | 10 (20.8) |
|  | DM+HTN+CAD | 6 | | 1 (16.7) | | 4 (66.7) | 1(16.6) |
|  | DM+HTN+CKD | 7 | | 2 (28.6) | | 4 (57.1) | 1 (14.3) |
|  | HTN+CAD | 2 | | 1 (50.0) | | 0 (0.0) | 1 (50.0) |
|  | HTN+CKD | 3 | | 1 (33.3) | | 1 (33.3) | 1 (33.3) |
|  | P value |  | 262 |  |  |  | 0.143 |

With increasing comorbidities, there is a significant increase in mortality, hospitalization, and severity in the cohort 1 table, which contrasts with cohort 2. Only the number of comorbidities determined the length of hospital stay, as evident from supplementary table 4 below. The length of hospital stay was significantly longer in patients having any three comorbidities compared to those without any, with one and two comorbidities, consistent with our cohort 1 data. The variations can be possibly attributed to evolved COVID-19 care management initiation of vaccination in the country.

Supplementary table 4. Overall survival, length of hospital stay and severity of the disease in COVID-19 patients

with multiple comorbidities

|  | **Number of comorbidities** | **Total** | **Survivors**  **(N, % within total)** | **Non- Survivors**  **(N, % within total)** | **Length of hospital stay**  **(in days)** | **95%CI** | **Mild**  **(N, % within total)** | **Moderate (N, % within total)** | **Severe (N, % within total)** |
| --- | --- | --- | --- | --- | --- | --- | --- | --- | --- |
|  | 0 | 133 | 129 (97.0) | 4 ^a^ (3.0) | 3.11^a^±2.47 | 2.69-3.54 | 72 (54.1) | 40 (30.1) | 21 ^a^ (15.8) |
|  | 1 | 63 | 61 (96.8) | 2 ^a^ (3.2) | 3.46^a^±2.57 | 2.81-4.11 | 28 (44.4) | 25 (39.7) | 10^a^ (15.9) |
|  | 2 | 55 | 51 (92.7) | 4 ^a^ (7.3) | 2.60^a^±2.02 | 2.05-3.15 | 24 (43.6) | 18 (32.7) | 13^a^ (23.6) |
|  | 3 | 14 | 12 (85.7) | 2 ^a^ (14.3) | 5.71^b^±3.64 | 3.61-7.82 | 4 (28.6) | 8 (57.1) | 2^a^ (14.3) |

In summary, variations existed in our data from cohort one and cohort two concerning the survival and severity of the disease.
